# RNA Modifications Modulate Activation of Innate Toll-Like Receptors

**DOI:** 10.3390/genes10020092

**Published:** 2019-01-29

**Authors:** Isabel Freund, Tatjana Eigenbrod, Mark Helm, Alexander H. Dalpke

**Affiliations:** 1 Department of Infectious Diseases, Medical Microbiology and Hygiene, Heidelberg University Hospital, 69120 Heidelberg, Germany; isabel-freund@t-online.de (I.F.); tatjana.eigenbrod@med.uni-heidelberg.de (T.E.); 2 Institute of Pharmacy and Biochemistry, Johannes Gutenberg-University Mainz, 55128 Mainz, Germany; mhelm@uni-mainz.de; 3 Institute of Medical Microbiology and Hygiene, Technical University Dresden, 01307 Dresden, Germany

**Keywords:** innate immunity, methylation, RNA modifications, Toll-like receptors

## Abstract

Self/foreign discrimination by the innate immune system depends on receptors that identify molecular patterns as associated to pathogens. Among others, this group includes endosomal Toll-like receptors, among which Toll-like receptors (TLR) 3, 7, 8, and 13 recognize and discriminate mammalian from microbial, potentially pathogen-associated, RNA. One of the discriminatory principles is the recognition of endogenous RNA modifications. Previous work has identified a couple of RNA modifications that impede activation of TLR signaling when incorporated in synthetic RNA molecules. Of note, work that is more recent has now shown that RNA modifications in their naturally occurring context can have immune-modulatory functions: Gm, a naturally occurring ribose-methylation within tRNA resulted in a lack of TLR7 stimulation and within a defined sequence context acted as antagonist. Additional RNA modifications with immune-modulatory functions have now been identified and recent work also indicates that RNA modifications within the context of whole prokaryotic or eukaryotic cells are indeed used for immune-modulation. This review will discuss new findings and developments in the field of immune-modulatory RNA modifications.

## 1. Introduction

Detection of pathogens by the innate immune system relies on a small subset of germline-encoded pattern recognition receptors (in contrast to re-arranged receptors of the adaptive immune system) that recognize structurally conserved microbial molecules. Beside cell wall components of bacteria and fungi, nucleic acids (NAs) have been identified as potent stimuli initiating a robust immune response. As both ‘self’ and ‘non-self’ nucleic acids are composed of the same basic building blocks, the ability to identify the origin of DNA and RNA is a crucial necessity for the innate immune system. Beside cytosolic receptors including amongst others retinoic acid inducible gene I (RIG-I), melanoma differentiation antigen 5 (MDA-5), absent in melanoma 2 (AIM2) and cyclic GMP-AMP synthase (cGAS), NAs are recognized by the Toll-like receptors (TLRs) 3, TLR7, TLR8, TLR9, and TLR13 which reside in the endosome. Numerous excellent reviews [1,2,3,4] have addressed ligand recognition by cytosolic receptors, thus the main focus of the present review will be NA sensing by pattern recognition receptors (PRRs) and the implications of RNA modifications in this context. 

Three main principles have been described that allow the innate immune system to avoid recognition of host (‘self’) NAs but allow stimulation by microbial (‘foreign’) NAs: First, discrimination can be based on spatial restriction of NA-sensing receptors to specific subcellular compartments. Thus, TLRs reside in the endolysosome to which self-RNA/DNA has no or only limited access. In contrast, microbial RNA/DNA can get access after endosomal uptake and degradation of microbes specifically in phagocytes. Second, nucleotide composition, naturally occurring chemical modifications, and distinct sequence motifs affect primary, secondary and tertiary structures of RNA and DNA and determine NA recognition. As such, 5’-triphosphorylated cytosolic RNA that arises during RNA-virus replication activates cytosolic RIG-I whereas eukaryotic “self” RNA avoids recognition through capping [5]. Unmethylated CG frequency is increased in prokaryotic as compared to mammalian DNA and thus is a pattern for TLR9 stimulation. The third principle to protect from self-recognition of especially host DNA is the degradation of nucleic acids by nucleases. One example is the cytosolic DNase III which prevents the accumulation of endogenous DNA and therefore the activation of cytosolic DNA sensors. Whereas under physiological conditions recognition of self-NAs is efficiently inhibited, self/non-self discrimination can fail in specific diseases. Of note, in a variety of autoimmune diseases it has now been reported that innate immune recognition of host NAs can facilitate onset and development of autoimmune reactions, e.g., in systemic lupus erythematosus and psoriasis [6,7,8]. Failures in any of the three described principles can thus result in autoimmunity and therefore a closer understanding of the molecular principles underlying NA recognition bears therapeutic options.

## 2. Activation of Endosomal Toll-Like Receptors by Nucleic Acids 

Endosomal NA-sensing TLRs are synthesized in the endoplasmic reticulum (ER) and shuttle via the Golgi apparatus to endosomes by the help of uncoordinated 93 homolog B1 (UNC93B1) [9]. In a recent study, Pelka et al. demonstrated that UNC93B1 is not only involved in trafficking but also expression and stabilisation of endosomal TLRs [10]. Ectodomains of shuttled immature TLRs are proteolytically cleaved within the acidic endosome [11,12,13,14]. Proteolytic cleavage of endosomal TLRs but not of TLRs localized at the cell surface is necessary for downstream-signaling. Of note, as NA-sensing TLRs are shuttled via the cell surface to the endosome, processing of immature TLRs by cleavage only at their final place of destination might be a further safety mechanism to prevent recognition of host DNA and RNA at the cellular surface (e.g., released DNA nets from neutrophils, NAs from apoptotic cells) [15]. 

DNA containing unmethylated CpG dinucleotide motifs which are common in bacterial DNA are recognized by TLR9 [16]. Interestingly, human and mouse TLR9 favor the two different CpG sequence motifs, GTCGTT and GACGTT, respectively [17]. Two classes of synthetic TLR9 ligands are described initiating different types of innate immune response: CpG-A desoxyoligoribonucleotides (ODNs) induce secretion of high amounts of interferon alpha (IFN-α) and maturation of plasmacytoid dendritic cells (pDCs), whereas CpG-B ODNs facilitate activation of B-cells but weakly stimulate IFN-α secretion in pDCs [16,18]. Whereas endosomal DNA is exclusively recognized by TLR9, RNA is sensed via TLR3, TLR7, and TLR8 in humans and TLR13 in mice. TLR3 recognizes the ribose-phosphate backbone of dsRNA without specific sequence requirements and binding of dsRNA larger than 40 base pairs results in the secretion of IFN-β [19,20]. While ssRNA is sensed by TLR7 and TLR8 in humans, in mice TLR7 and TLR13 are the relevant receptors [21]. TLR7 and TLR8 are similar in amino acid sequence and biological function but differentially expressed among various cell types. Activation of TLR7 in plasmacytoid dendritic cells (pDCs) results in the release of high amounts of type I interferons, whereby stimulation of TLR8 in monocytes and macrophages predominantly produce proinflammatory cytokines like interleukin-6 (IL-6), IL-12p70, and tumor necrosis factor (TNF) [22,23,24]. Both receptors have been shown to sense ssRNA and small chemical imidazoquinoline components like Resiquimod (R848), however, TLR7 is discussed to recognize also dsRNA [25]. Recently, crystal structures of the ectodomains of TLR7 and TLR8 dimers bound to their specific ligands have been identified [26,27]. For both TLRs two different binding sites were detected. Interestingly, the first binding site sensing small chemical components seems to be conserved among the receptors. The recognition site is localized in the dimerization interface of the receptors and upon ligand binding dimerization is induced. Of note, the second binding site of TLR7 and TLR8, which is responsible for RNA sensing, differs regarding sequence specificity and localization. The second binding site of TLR7 binds a polyU 3-mer and is located in the dimerization interface, whereas the second binding site of TLR8 is localized outside the dimerization interface and senses UG or UUG oligoribonucleotides. Interestingly, the first binding site of TLR7 and TLR8 detecting small chemical ligands is also involved in RNA sensing by binding a single guanosine or uridine, respectively. Moreover, crystal structures encourage previously described preferential recognition of GU-rich motifs by TLR7 and TLR8 [25].

Interestingly, recognition of ssRNA differs between mice and humans, as TLR8 has limited function in mice and TLR13 is not present in humans [28,29]. Of note, TLR13 is unique among other RNA-sensing receptors as it exclusively senses a highly conserved sequence within the 23S ribosomal RNA of bacteria and therefore shows strong substrate specificity [30,31]. Oldenburg et al. demonstrated that N^6^ methylation at A2085 of *Staphylococcus aureus* 23S rRNA (corresponding to A2058 of *E. coli* 23S rRNA) which also induces macrolide resistance of certain bacteria abolishes TLR13 stimulation [31]. The latter modification thus not only induces antibiotic resistance but also decreases immune-recognition, an interesting dual function. 

## 3. RNA Recognition by the Innate Immune System Indicates Infectious Danger

Recognition of ssRNA by human TLR7 and TLR8 has been shown since years to be important to mount antiviral responses. However, whether RNA recognition also plays a role in bacterial infection remained underexplored. Of note, several studies in recent years now elucidated the importance of bacterial RNA recognition for innate immune response [21,32,33,34]. Rapid degradation of NAs upon microbial cell death makes RNA a potential hallmark to discriminate viable bacteria from less harmful dead bacteria by the innate immune system [35]. A concept was suggested claiming that innate immunity should be able to discriminate viable from dead microbes, the first ones representing a higher level of infectious danger and thus should result in a qualitatively and quantitatively different response. Microbial patterns that fulfill these criteria have been named vita-PAMPs (vitality pathogen associated molecular patterns). ssRNA, especially prokaryotic mRNA, is such an example and is recognized by TLR7 and TLR8 within pDCs and monocytes, respectively. Stimulation of TLR7 results in the secretion of high amounts of type I interferons which are inducing the production of so called interferon-stimulated genes (ISGs) impeding viral replication but also playing a role in bacterial defense and proinflammatory cytokines facilitate production of reactive oxygen and nitrogen species and promote phagocytosis to limit bacterial infection [36,37,38]. 

Recognition of foreign RNA in the cytosol is generally considered as hallmark of viral infection and replication [39]. Yet, even for microbes replicating in the cytosol, endosomal TLRs might play a role, as it has been shown that autophagy enables sensing of cytosolic PAMPs in the endosomal compartment [40,41]. Thus, the sub-cellular compartmentalization of nucleic acids might be not as strict as thought. During viral infection, autophagy seems to be induced to improve IFN production, however, TLR signaling is also described to negatively regulate autophagy to control interferon response [42]—perhaps as a negative regulatory circuit. The usual access of endosomal TLRs to their NA ligands is by endosomal uptake of extracellular microbes. Thus, RNAs taken up by endocytosis of living bacteria or viruses are delivered to the endosome and are recognized by TLR3, TLR7, and TLR8 [43,44]. RNA recognition is associated with detection of “viability”, indicating the presence of harmful bacteria and in consequence provokes secretion of high amounts of IFN-β and activation of the so-called inflammasome, a multi-protein complex culminating in the self-cleavage of caspase-1 with subsequent generation of bioactive IL-1β. Just recently, Ugolini et al. demonstrated that TLR8-dependent recognition of living bacteria through their RNA induces a specific cytokine profile that amongst other cytokines was peculiar for high IL-12 induction [45]. Only RNA-activated human and porcine antigen presenting cells (APCs) were able to generate a downstream cytokine response facilitating robust antibody production. Thus, RNA recognition by the innate immune system also affects secondary stimulation of adaptive immunity. Furthermore, the authors demonstrated higher levels of immunoglobulins after vaccination with living compared to dead bacteria, confirming RNA as a potent co-stimulus for antibody formation [45]. Consonantly, Ziegler et al. identified a single stranded polyU-rich RNA as potent adjuvant for vaccinations to induce high amounts of specific antibodies [46]. 

However, RNA recognition by the innate immune system is not always beneficial. Sensing of self NA can trigger IFN and proinflammatory cytokine-associated severe autoimmune diseases like systemic lupus erythematosus (SLE) or psoriasis [47,48,49]. Endogenous nucleic acids complexed with high mobility group box chromosomal protein (HMGB) 1, heat-shock protein 90 or the antimicrobial peptide LL37 stimulate type I interferon producing pDCs [50,51,52,53]. Moreover, mutations in genes related to autophagy are associated with SLE [54]. In two recent publications Weindel et al. demonstrated that loss of B-cell autophagy prevents SLE symptoms in mice, indicating autophagy is required for disease progression. However, Tlr7.1 transgenic mice lacking autophagy in both, B-cells and DC developed lethal inflammatory condition similar to sterile sepsis, suggesting that autophagy plays a dual role for cytokine signaling in vivo [55,56]. Furthermore, cutaneous lupus erythematosus (CLE) is associated with IFN-driven inflammation induced by the recognition of endogenous NAs. Scholtissek et al. verified the capacity of self RNA and DNA released by dying cells to induce secretion of proinflammatory cytokines in keratinocytes and to maintain inflammatory processes in CLE skin lesions [57]. Thus, recognition of endogenous NAs under pathophysiological conditions is a main reason of enhanced IFN response of the innate immune system in autoimmune diseases. However, under homeostatic conditions, endogenous NA recognition is reliably prevented. The principles of discrimination are just now being deciphered.

## 4. Mechanisms to Avoid Self DNA/RNA Recognition: Degradation and Modification

Limitation of endogenous DNA and RNA recognition by spatial restriction of NA-sensing TLRs to the endolysosome, degradation, and discrimination by sequence motifs and chemical modifications are secure strategies to impede self-recognition.

Why are TLRs recognizing nucleic acids restricted to the endosome, whereas other NA-sensing PRRs are localized in the cytosol? First of all, host DNA is not expected to be freely available in the cytosol. However, accumulation of endogenous DNA derived from viral retroelements is impeded by the cytosolic DNase three-prime repair exonuclease 1 (TREX1 or DNase III). Thereby, degradation of DNA within the cytosol negatively regulates cGAS-STING signaling and prevents IFN production [58]. However, mutations in the lysosomal DNases II impede recognition of DNA by endosomal TLR9. Recently, Chan et al. demonstrated that digestion of CpG-A is required for TLR9 and DC activation in vitro [59]. However, a biallelic loss-of-function mutation in the DNASE2 gene causes autoinflammatory state and enhanced type I interferon signaling in humans. DNase II deficiency-based DNA-overload within the endolysosome and self-DNA leakage to the cytosol facilitates activation of cGAS promoting IFN secretion [60]. Furthermore, mouse studies suggested the responsibility of macrophages for autoinflammatory state and arthritis symptoms by secretion of high level of TNF, IL-1β, and IL-6 in case of DNase II deficiency [61]. Thus, self-DNA-recognition is mainly prevented by DNA digestion.

**Figure 1:**
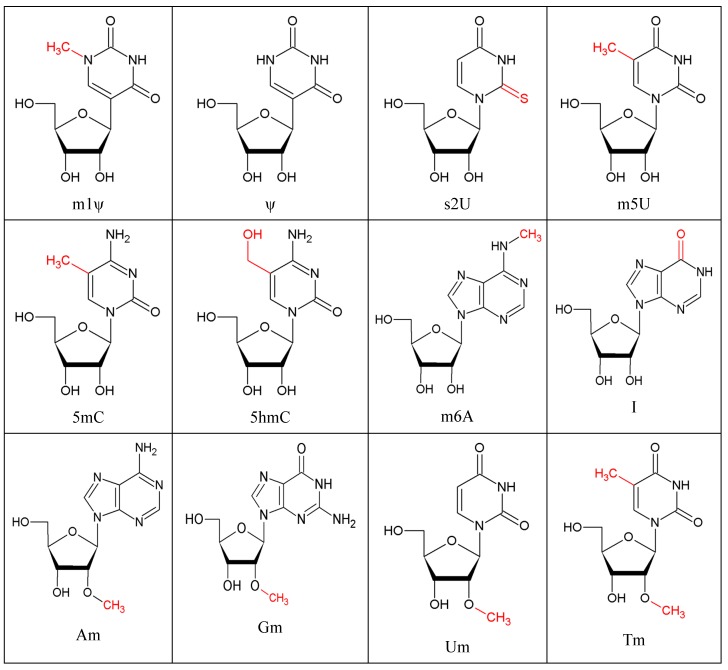
Selected RNA modifications with immune-modulatory properties Structures correspond to Table 1 and refer to [91] 2’-*O*-methylation of RNA in a physiological sequence context.

In contrast, recognition of self-RNA is especially avoided by RNA modifications. More than 150 different, naturally occurring posttranscriptional RNA modifications are meanwhile identified [62]. Eukaryotic RNA in general is more heavily modified compared to prokaryotic RNA and RNA modifications can be notably more complex thus allowing a potential discrimination of the evolutionary origin by the innate immune system. In a seminal paper, Karikó et al. elucidated differences in immune stimulation of RNA dependent on its evolutionary origin [63]. The authors reported a negative correlation of immune stimulatory potential of RNA and extent of incorporated modifications. Based on the basic observation that secretion of innate cytokines was inefficient with mammalian cytosolic RNA, but efficient with bacterial RNA as well as mitochondrial RNA, Karikó et al. specifically analyzed activation of TLR3, 7, and 8 by modified RNA. In vitro transcribed RNA containing randomly incorporated s2U or m6A lacked TLR3 stimulation. m6A, m5C, m5U, s2U, and Ψ modifications impeded stimulation of TLR7 and 8 (see Figure 1 for structures). The latter modifications also inhibited secretion of TNF and IL-12 from monocyte-derived dendritic cells. However, in primary DC populations m6A and m5C modified RNA was stimulatory with respect to TNF secretion indicating cell type differences. Of note, when RNA with Ψ was given in parallel, immunostimulation decreased indicating that some RNA modifications might actively inhibit recognition of stimulatory RNA. In general, immune stimulatory potential of different total RNA samples depends on their evolutionary origin. RNA derived from bacteria is more immune stimulatory compared to human RNA. Bacterial ssRNA associated GU rich motifs are recognized by TLR7 and TLR8 [64,65]. At a structural level it was shown that TLR8 recognizes two degradation products of RNA at two different sites [27]: Site 1 was bound by uridine at the TLR dimerization interface. This is also the sight where small chemical ligands bind to TLR8. However, a second site on the concave surface of the horseshoe shaped extracellular domain of TLR8 was occupied by a short oligonucleotide. Binding preferences were in accordance with recognition of uridine- and guanosine-rich ssRNA as reported before, yet surprisingly the interaction apparently took only place with small RNA degradation products. Crystal structure analysis of TLR-7 also revealed two binding sites within the m-shaped dimer. A first site was conserved in TLR7 and TLR8 and bound small ligands, preferentially guanosine. A second binding site, different from the TLR8 structure, interacted with ssRNA which enhanced affinity of the first site for its ligand. For the second sites uridines were preferred. The authors suggested that TLR7 is a dual receptor recognizing guanosine and ssRNA. In turn the data would suggest that RNA has to be degraded prior to interaction with TLR7/8 and this might be a molecular point of action for RNA modifications.

Recently, incorporation of *N*(1)-methylpseudouridine into mRNA, either alone or in combination with m5C, was shown to further decrease stimulation of innate immunity, especially TLR3 as compared to pseudouridine [66]. As mRNA stability in parallel was improved, this modification was suggested to have increased performance for mRNA-based therapeutics. Indeed it was shown that in vivo a *N*(1)-methylpseudouridine modified mRNA vaccine induced better T-follicular helper cell responses and germinal B cell activation—yet it remained unclear whether this was due to effects on mRNA stability or reduced innate immune stimulation [67].

## 5. RNA Modifications Affecting Innate Immune Receptors Other than TLRs

Besides RNA modifications affecting stimulation of TLRs, the same principle also holds true for other innate immune sensors. As a brief overview, the following findings have been made in the field. Polyadenylation in eukaryotic RNA limited induction of IL-12 from human monocyte-derived DCs, which was high upon activation by bacterial [68]. Confirming these findings, in-vitro transcribed mRNA lacking a poly(A) tail induced immune activation and this could be abolished by enzymatic 3’-polyadenylation. 

Of utmost importance, cytosolic sensing of self-RNA by RIG-I or MDA-5 is prevented by specific modifications impeding RNA recognition. A to I conversion by the enzyme adenosine deaminases acting on RNA (ADAR) is one important strategy to make host RNA invisible for the innate immune system [69,70]. This topic has gained much interest in the last years and has been reviewed elsewhere [71,72]. Indeed, adenosine deamination is the most common modification affecting innate sensing. In brief, ADARs hydrolytically deaminate adenosines in regions of duplex RNA, whereby inosine mimics guanosine in hydrogen bonding and RNA function can be significantly modified. Mannion et al. were the first to connect ADAR activity to innate immune sensing: ADAR1 mutations cause Aicardi-Goutières syndrome associated with increased interferon expression. *Adar1* mutant mice die by embryonic day E12.5 and show aberrant interferon production. Yet, mice mutant for *Adar1* and *Mavs*, the latter being the main signal adaptor for RIG-I and MDA5, survived thus showing that the phenotype probably is linked to recognition of unmodified dsRNA by cytosolic innate sensors. The rescuing effect on interferon production was dependent on the editing activity [69]. In the same line, Liddicoat et al. also identified A-to-I editing activity of ADAR1 to be crucial for embryonic development in vivo [70]. Transcriptional profiling of mice deficient for ADAR1 elucidated the up-regulation of 383 transcripts (log2FC > 2), whereby two thirds of those transcripts (258) were IFN-stimulated genes (ISGs). The authors concluded self-recognition of mouse dsRNA by MDA-5 or RIG-1. Interestingly, double knock-out of ADAR1 and MDA-5 in mice rescued embryonic development and mice were only slightly smaller than littermate controls without demonstrating additional phenotypes. ADAR1 p150 editing in murine fibroblasts was also suggested to act as feedback suppressor mechanism during innate immune responses, as interferons increased editing, thereby limiting recognition of self-RNAs with double-stranded portions [73]. The data impressively demonstrated importance of A-to-I editing for self/non-self discrimination of RNA by cytosolic PRRs. However, the exact mechanism of action is not entirely known, e.g., it was suggested that ADAR rather acts by RNA binding than by editing [74].

Moreover, RNA transcribed in the nucleus is modified with a 5’ cap. This is characterized by an inverted 7-methylguanosine linked the first transcribed nucleotide (5′ cap) and 2’-*O*-ribose methylation of the first (cap 1) and often the second (cap 2) nucleotide which abolishes interaction with RIG-I and MDA5 [5,75]. In contrast, RNA formed outside the nucleus by viral replication exhibits a 5’ triphosphate which is recognized by RIG-I. Hence, through evolutionary processes viruses like flaviviruses, coronaviruses and poxviruses have developed alternative 5’-elements including 2’-*O*-methylation avoiding recognition by the innate immune system [76]. Structurally, it was shown that a single conserved amino acid (H830) in the RIG-I RNA binding pocket was responsive for steric exclusion of *N*1-2’-*O*-methylated RNA and thus contributes to self/foreign discrimination [5]. However, it has also been shown that 2’-*O*-methylation of the viral mRNA cap in West Nile Virus is important to restrict the actions of antiviral IFN-induced proteins with tetratricopeptide repeats (IFIT) resulting in loss of pathogenicity in a mutant absent of 2’-*O*-methyltransferase activity [77,78]. Thus, RNA modifications might not only affect recognition of RNA (and subsequent induction of innate cytokines) but also might regulate sensitivity towards innate immune effector molecules. Besides 2’-*O*-ribose methylation of the cap, NS5 protein of flaviviruses might also act as methyltransferase for internal adenosines thus extending the amount of modified nucleotides and thereby eventually reducing immunostimulation [79]. Besides inhibition of RIG-I by 2’-*O*-methylation it was also shown that MDA5 activation is impeded by 2′-*O*-methylation in viral mRNA: Coronavirus mutants lacking 2′-*O*-methyltransferase activity showed increased type I interferon production and were also more susceptible to IFN actions [80]. Moreover, *N*-6-methyladenosine (m6A) and pseudouridine within a polyU/UC RNA domain of hepatitis C virus were tested for cytosolic RIG-I stimulation and showed to reduce RIG-I signaling. Similar modifying effects were also seen for 2FdU, 2FdC, 5mC, 5moC, and 5hmC. However, while m6A modified RNA bound RIG-I poorly, the mode of action was different for pseudouridine: this had high affinity for RIG-I but failed to induce a conformational change necessary for activation [81].

Preferential recognition of RNA is based on sequence composition and RNA modifications. Several studies investigated the immunosilencing capacity of ribose-methylation within ssRNA. In this regard, a single 2’-*O*-methylation of guanosine within certain tRNA isoacceptors was described to prevent RNA from TLR7 and TLR8 activation. This *cis*-silencing (which means the modified RNA itself is not recognized) properties of ribose-methylation are used to modify siRNA to avoid immune recognition [24,92,93]. Moreover, ribose 2’-*O*-methylation is known to act as a dominant inhibitor on TLR7 and TLR8, which means 2’-*O*-methylation of only a proportion of RNAs is sufficient to abrogate immune stimulation of the entire RNA preparation. Mechanistically, it was shown that 2’-*O*-methylated RNA competed with unmodified RNA for TLR7 binding [94]. At the signaling level early activation events, e.g., MAP kinase activation were inhibited. It was suggested that 2’-*O*-methylated RNA might displace non-methylated stimulatory RNA from its receptor without inducing the change in TLR7 conformation that is necessary for activation. Supporting the same argumentation, 2’-*O*-methylated RNA was shown by AlphaScreen™ to bind with higher affinity to TLR7 compared to unmodified control, which might explain the *trans*-silencing effect (that is otherwise immune stimulatory RNAs are not recognized in the presence of 2’-*O*-methylated RNA) properties of RNA 2’-*O*-methylation) [89].

In its physiological sequence context, 2’-*O*-methylation of G18 within tRNAs is described to stabilize their L-shaped three-dimensional structure. In this regard, extremophilic bacteria tend to exhibit more excessively modified tRNA [95,96]. Most enzymes causing posttranscriptional RNA modifications are site and/or sequence specific. Therefore, chemically identical modifications at different positions within tRNAs require specific enzymes [97]. The bacterial tRNA-2’-*O*-methyltransferase trmH, which is responsible for 2’-*O*-methylation of conserved guanosines at position 18 (Gm18) in the D-loop of tRNAs, is one example for diversity of enzymes. Based on the substrate specificity, trmH enzymes can be assigned to two classes: type I *trmH* methylates all tRNA species at G18, whereas type II *trmH* only modifies a subset of tRNA isoacceptors. Homologues of *trmH* are characterized in lower as well as higher eukaryotes. For instance, in *Saccharomyces cerevisiae* the tRNA-2’-*O*-methyltransferase is called *trm3* and in humans it is termed TARBP1 [97,98,99,100]. Besides pseudouridine, 2’-*O*-methylation is one of the most abundant posttranscriptional modifications within eukaryotic ribosomal RNA. Up to one hundred 2′-*O*-methylation sites within yeast or human rRNA are known and modified by nuclear protein 1 (NOP1) or fibrillarin, respectively [101,102]. Those methyltransferases are described to be crucial for cell viability and—among other diverse functions—fibrillarin is participating in early pre-rRNA processing and modification and ribosome [103,104]. rRNA 2’-*O*-methylation is supporting ribosomal structures and therefore seems to be essential for stability and function [105]. Of note, recently it was shown that tRNA methyltransferase TRMT61B also acts on mitochondrial 16S rRNA within vertebrates and induces 1-methyladenosine modification. Thus, enzymes seem to exist that modify both, tRNA and rRNA. Whether this affects immunogenicity of the RNA is currently unclear [106]. In general, diversity of functions and host specific localization renders RNA modifications to an essential feature of immune modulation, RNA stabilization and cell viability.

## 6. Immune-Modulating Effects of RNA Modifications in the Natural Sequence Context 

Initially, 2’-*O*-methylation of siRNA was used to improve stability of synthetic siRNAs in the serum [107]. However, in 2005 Morrissey et al. identified 2’-*O*-methylation of siRNA to inhibit immune stimulation, a common undesired side-effect of siRNA application [93]: The authors demonstrated the absence of IFN-α, IL-6 and TNF when modified siRNA was applied in vivo. Immune suppressing effects on TLR7 using synthetic 2’-*O*-methylated RNA in human as well as murine plasmacytoid DCs and monocytes were confirmed then in a number of studies [17,108,109]. Those findings are in line with previous observations that dsRNA-homopolymers containing 2’-*O*-methylation induced less type I IFN.

As another key finding, Robbins et al. identified 2’-*O*-methylated RNA as TLR7 antagonist [82]. Interestingly, the authors demonstrated inhibition of TLR7 by 2’-*O*-methylated RNA in *trans*, which means TLR7 signaling was impeded when only one strand of the siRNA duplex was modified. The data argued for a “dominant” inhibitory effect of 2’*O*-methylated RNA over otherwise immune-stimulatory RNA. To examine whether inhibition requires annealing of modified and unmodified RNA, 2’-*O*-methylated RNA was encapsulated together with non-complementary immune stimulatory RNA. Of note, co-delivery of non-complementary modified and unmodified RNA at a 1:1 ratio efficiently abrogated immune stimulation of PBMCs. Yet, early data with 2’O-methylation made only use of synthetic RNA.

2’-*O*-methylation however is also naturally occurring at significantly higher abundance in eukaryotic as compared to prokaryotic (and mitochondrial) RNA [110,111] and thus the findings were indicative of a concept of recognition of “hypomethylated” RNA to discriminate self/foreign origin.

Immune modulatory potential of 2’-*O*-methylation in its physiological context was described for the first time within tRNAs. In 2012, Jöckel et al. and Gehrig et al. described the immune silencing effect of 2’-*O*-methylation of guanosine at position 18 (Gm18) of tRNAs. Gm18 within *E. coli* tRNA was sufficient to abolish immune stimulation of certain isoacceptors as well as total tRNA fractions [64,85]. It was also shown that a single 2’*O*-methylation within a self-RNA is able to interfere with TLR stimulation thus hinting towards a physiological function in self/foreign discrimination. Interestingly, also in in this study the suppressive activity, when 2’*O*-methylation was placed in a different than the immune stimulatory RNA strand, was confirmed [112]. 

Besides 2’-*O*-methylation, 2’-*O*-fluoro modification has also been shown to limit inflammatory responses by therapeutic RNAs [88]. Several studies described immune inhibition of 2’-*O*-methylated guanosine, adenosine and uridine but ribose-methylation of cytosine failed to inhibit TLR7 or TLR8 response [24,82]. However, in the latter studies the sequence context of 2’-*O*-methylation-dependent immune inhibition was not elucidated. Thus, Kaiser et al. performed a permutation study of 2’-*O*-methylated nucleotides also considering the nucleobase downstream of the modification [84]. The authors elucidated a functional (DmR) motif (D = all but C; R = purine) which inhibited TLR7 stimulation in *cis*. However, a sequence motif inhibiting TLR8 stimulation was pending. Furthermore, Kaiser et al. only focused on TLR inhibition by 2’-*O*-methylation in *cis* but did not verify if identified immune inhibitory sequences act as TLR7/8 antagonists in *trans.* Co-transfection experiments with immune inhibitory 2’-*O*-methylated oligoribonucleotides (ORNs) and immune stimulatory bacterial RNA performed by Schmitt et al. characterized the two dominant inhibitory tri-nucleotide motifs (*D*m*R*C) motif (D = all but C; R = G, A) and (*D*m*DN*) motif (D = all but C; R = G, A; N = all) inhibiting TLR8 and TLR7 in *trans*, respectively [83]. Interestingly, the motif differed between TLR7 and TLR8, whereby immune inhibitory nucleotide sequence seemed to be more stringent for TLR8 compared to TLR7. Even more surprising, the nucleobase two positions downstream of the 2’-*O*-methylated nucleotide was most stringent for TLR8 inhibition. Previous findings of Jung et al. suggested that a single 2’-*O*-methylation within a synthetic 18S rRNA derived sequence can abolish TLR7 activity while retaining TLR8 stimulation [113]. This observation is supporting the two discriminative tri-nucleotide motifs. Given the slightly different inhibitory sequence motifs for TLR7 and TLR8 as identified by Schmidt et al., it is probable that this “specificity” is not a general effect but very much dependent on the specific sequence context of the modification [83]. Indeed, Jung et al. used a GmGU motif which would inhibit TLR7 (DmDN) but not TLR8 (DmRC). Moreover, Schmitt et al. identified a minimal length of 9 nucleotides containing the intact tri-nucleotide motif at position 1–7 as most efficient ORN size to abrogate TLR8 activation. Moreover, recent studies indicated a shielding effect of bulky nucleotide modifications on TLR7 recognition [87]. Those kinds of modification seem to impede TLR signaling by steric shielding of the RNA towards the receptor in *cis* without inhibiting TLR7 in *trans*. Interestingly, those modifications did not exhibit a discernible common term of chemical structures. Steric shielding of RNA was introduced by several kinds of modifications like small molecules, sugar derivates or fluorescent dyes. 

## 7. Silencing versus Antagonizing Modifications

Dominant inhibition of TLR7 by 2’-*O*-methylated RNA has been acknowledged for many years. Interestingly, the native human tRNA^Lys^_3_ was described as non-stimulatory on the innate immune system without exhibiting previously described 2’-*O*-methylation at position 18. Co-transfection experiments with otherwise immune stimulatory bacterial RNA elucidated that tRNA^Lys^_3_ itself is indeed non-stimulatory without inhibiting TLR7 response against stimulatory RNA. A double-methylation of uridine to 2’-*O*-methyl thymidine at position 54 of human tRNA was identified as sufficient to partially silence immune recognition of RNA without dominant inhibition of TLR7 [86]. However, this effect was considerably weaker compared to Gm18 indicating that the collective engagement of all posttranscriptional modifications within human tRNA^Lys^_3_ contribute to its immune silencing effect. Moreover, previously described shielding effects of bulky RNA modifications which were also found in the anticodon of human tRNA^Lys^_3_ might reinforce immune silencing effects of the RNA. 

**Figure 2:**
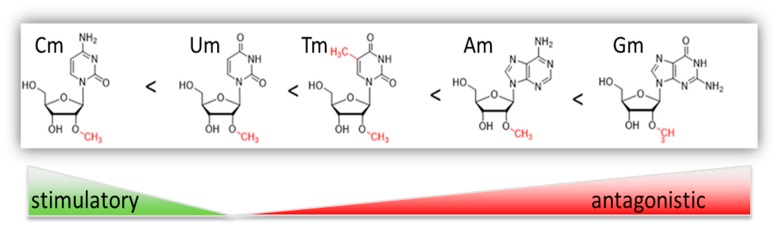
Immune modulatory properties of 2’-*O*-methylated nucleotides. Increase of nucleotide structure shifts 2’-*O*-methylated immune stimulatory nucleotides (cytidine) towards immune antagonistic nucleotides (adenosine, guanosine).

Altogether, data suggest a significant influence of the nucleobase on dominant inhibition of TLR7 and TLR8. Recent insight on 2’-*O*-methythymidine closed a gap in the understanding from inert to immune silencing to antagonistic RNA modifications based on 2’-*O*-methylation. Increasing structure of the nucleobase seems to shift immune stimulatory properties of a pyrimidine base to immune inhibitory features of a purine base (Figure 2).

Recently, Hu et al. tested RNA modifications to get insight into the mechanism of TLR8 activation. Based upon the crystal structure of TLR8 bound to ssRNA two RNA interaction sites had been identified with one site recognizing a UpG dinucleotide. Applying atomic mutagenesis RNA analogues containing 7-deazaguanosine, 2-aminopurine and inosine confirmed the function of N7, O6 and N2 for TLR8 activation. Of note, in such a way modified oligoribonucleotides however retained their ability to antagonize TLR8 activation by RNA [114]. It had been shown that 2’-*O*-methylation only affects stimulation of TLR7/8 by RNA but not by small molecule ligands [94]. Thus, it is probable that antagonistic activity e.g., of 2’-*O*-methylated RNA is due to interference a bindings site 2 within TLR7/8 as this site is not occupied by small molecule ligands.

## 8. Importance of RNA Modifications for Immune Regulation In Vivo

Mutations in nucleic acid modifying enzymes are associated with severe diseases including cancer, asthma and mitochondrial dysfunctions [115]. Moreover, RNA methylation is linked to intellectual disability, indicating relevance of RNA modifications in the development of cognitive functions. As an example, mutations in the human *FTSJ1* gene cause lack of 2’-*O*-methylation of C32 and G34 of human tRNAs and are associated with non-syndromic X-linked intellectual disability [116]. Reduced A-to-I RNA editing levels in Alzheimer’s disease patients’ brain tissues, mainly in the hippocampus and to a lesser degree in the temporal and frontal lobes were recently associated with neurodegenerative processes [117]. Besides these findings, RNA mutations were also linked to altered immune functions supporting the concept of RNA modifications as innate immune regulators. As such, deaminase ADAR1 protects host dsRNA from recognition by the innate receptor MDA5. Of note, mutations in *ADAR* genes were linked to Aicardi-Goutières syndrome (AGS), a diseases which is associated with increased levels of IFN-α [69,118]. Thus, also in vivo, RNA modifications are linked to immune functions. 

2’-*O*-methylations in ribosomal RNAs are introduced by fibrillarin in humans [103]. Fibrillarin is implicated in early processing of pre-rRNA, ribosome assembly and cell growth [104]. Ribosomal structure, stability and function are dependent on 2’-*O*-methylation of rRNA [105]. Of note, anti-fibrillarin antibodies are frequently observed in the serum of patients suffering from rheumatic autoimmune diseases like systemic sclerosis (SSc), systemic lupus erythematosus (SLE), primary Raynaud’s phenomenon and myositis [119,120,121,122]. Rheumatic disease-associated inflammatory response has been linked to recognition of endogenous nucleic acids like RNA [123,124]. However, the impact of anti-fibrillarin antibodies on extend of rRNA methylation has not yet been investigated. Decrease in 2’-*O*-methylation within rRNA might affect RNA sensing by TLR7 and TLR8 and therefore could facilitate self-NA recognition and type I IFN secretion. The above described studies investigating the effect of 2’-*O*-methylation with regard to its immune silencing properties were mostly performed with isolated RNA species. Most often certain tRNA isoacceptors or whole tRNA preparations were used to elucidate the relevance of ribose methylation for immune recognition. However, the relevance of Gm18 within pro-and eukaryotic tRNAs in a more physiological context like during infections with certain bacteria or yeast has not been elucidated so far. Although eukaryotic rRNA is more heavily modified concerning 2’-*O*-methylation compared to prokaryotic rRNA, studies on the immune modifying relevance of ribose methylation are still pending. Knock-out of Nop1 in yeast (necessary for rRNA 2’*O*-methylation) is described as lethal [101], however, knock-down experiments of Fibrillarin in human HeLa cells by siRNA and decrease of methylation within rRNA were already successful [125]. Decrease of 2’-*O*-methylation within human rRNA facilitates on one hand the investigation of isolated rRNA with respect to immune stimulation and on the other hand, knock-down experiments of fibrillarin in human immune cells like pDCs or monocytes might answer the question if self-recognition of host RNA is facilitated by inappropriate ribose methylation.

## 9. Conclusions

The ability to posttranscriptionally modify certain nucleotides within DNA and RNA recently has been shown to have an additional function beside of structural needs and epigenetics which is marking NAs as self or non-self to allow immune recognition. Modified nucleotides not only reliably prevent self-NA recognition under homeostatic conditions but also modulate innate immune response. The broad repertoire of NA modifications allows a receptor specific inhibition of RNA or DNA recognition. However, the importance of modifications to regulate immune activation is a double-edged sword needing to balance adequate immune activation upon infection and self-tolerance. Misused RNA modifications by certain pathogens may cause immune evasion while inappropriate processing of self NAs can cause autoimmune diseases. Thus, the understanding of complex NA modification pathways and the impact of nucleotide modifications on the innate immune system will remain a challenging task that now enters the stage of studies under physiological and pathophysiological conditions. 

## Figures and Tables

**Table 1:** Overview of selected immune modulatory RNA modifications.

Modification	Receptor	Investigated Cell Type	Effect	Reference
ψ	RIG-I	Huh7 cells; luciferase reporter	decreased receptor activation	[81]
m1ψ	RIG-I	Huh7 cells; luciferase reporter	decreased receptor activation	[81]
m6A	RIG-I	Huh7 cells; luciferase reporter	reduced RNA signaling	[81]
2FdU	RIG-I	Huh7 cells; luciferase reporter	reduced RNA signaling	[81]
2FdC	RIG-I	Huh7 cells; luciferase reporter	reduced RNA signaling	[81]
5mC	RIG-I	Huh7 cells; luciferase reporter	decreased receptor activation	[81]
5moC	RIG-I	Huh7 cells; luciferase reporter	reduced RNA signaling	[81]
5hmC	RIG-I	Huh7 cells; luciferase reporter	reduced RNA signaling	[81]
5’terminal -2’-*O*-methylation	RIG-I	PBMCs	steric exclusion of RNA	[5]
I	RIG-I/MAVS	MEFs	A to I editing	[69,70]
5’terminal 2’-*O*-methylation	MDA5	human blood-derived macrophages	impeded RNA recognition	[80]
2’-*O*-Me	MDA5	Murine macrophages	Inhibition of type I IFN induction	[80]
I	MDA5/MAVS	HEK293T cells, MEFs	A to I editing; reduced RNA signaling	[69,70]
s2U	TLR3/7/8	HEK293 overexpression of TLR, MDDCs, primary Dendritic Cells (DCs)	reduced RNA signaling	[63]
m6A	TLR3/7/8/13	HEK293 overexpression of TLR, MDDCs	reduced RNA signaling	[31,63]
m5C	TLR7/8	HEK293 overexpression of TLR, MDDCs	reduced RNA signaling	[63]
m5U	TLR7/8	HEK293 overexpression of TLR, MDDCs, primary DCs	reduced RNA signaling	[63]
Ψ	TLR3/7/8	HEK293 overexpression of TLR, MDDCs, primary DCs	reduced RNA signaling	[63]
m1Ψ	TLR3	A549	reduced mRNA immune activation	[80]
polyA tail		MDDC	reduced mRNA immune activation	[68]
Am	TLR7/8	PBMCs	dominant inhibition of TLR signaling	[24,82,83,84]
Gm	TLR7/8	PBMCs	dominant inhibition of TLR signaling	[24,64,82,83,84,85]
Um	TLR7/8	PBMCs	dominant inhibition of TLR signaling	[24,64,78,79,80,81]
Tm	TLR7/8	PBMCs	reduced TLR signaling	[86]
Monomannose	TLR7	PBMCs	steric shielding	[87]
Trimannose	TLR7	PBMCs	steric shielding	[81]
2’-*F*, 2’*O*-Me	TLR3/7	PBMC	reduced RNA/miRNA/siRNA immune stimulation	[88,89,90]

MDDC, human monocyte-derived dendritic cells; PBMCs, human peripheral blood mononuclear cells; TLR: Toll-like receptor.
